# Integrated analysis of differentially expressed long noncoding RNAs and mRNAs associated with high-fat diet-induced hepatic insulin resistance in mice

**DOI:** 10.1186/s12986-020-00467-7

**Published:** 2020-06-18

**Authors:** Zengyuan Zhou, Xue Zhao, Liang Chen, Yuzheng Li, Zhao Chen, Yuanyuan Wang, Zihao Zhou, Xia Chu

**Affiliations:** Department of Nutrition and Food Hygiene, Public Health College, Harbin Medical University, 157 Baojian Road, Nangang District, Harbin, Hei Longjiang province 150081 P. R. China

**Keywords:** High-throughput sequencing, Long noncoding RNAs, Hepatic insulin resistance

## Abstract

**Background:**

Hepatic insulin resistance (IR) is an early pathological characteristic of many metabolic diseases, such as type 2 diabetes. Long noncoding RNAs (lncRNAs) have been identified as mediators of IR and related diseases. However, the roles of lncRNAs in hepatic IR remain largely unknown.

**Method:**

High-throughput sequencing was performed on ten liver tissue samples from five normal diet (ND)-fed mice and five high-fat diet (HFD)-induced hepatic IR mice, respectively. lncRNAs and mRNAs that were differentially expressed (DE) between the two groups were identified by bioinformatic analyses. Seven DE lncRNAs were validated by quantitative real-time PCR (q-PCR). The potential functions of the DE lncRNAs were predicted by Gene Ontology and Kyoto Encyclopedia of Genes and Genomes pathway analyses of target genes. In addition, integrated analysis was performed for the DE lncRNAs and mRNAs to predict their interaction relationships.

**Results:**

A total of 232 DE lncRNAs were identified in the HFD-induced hepatic IR mice compared with the ND-fed mice. These DE lncRNAs included 108 upregulated and 124 downregulated lncRNAs, and 7 of the DE lncRNAs were validated by q-PCR. In addition, 291 DE mRNAs including 166 upregulated and 125 downregulated mRNAs were identified in the HFD group. Furthermore, target genes of the DE lncRNAs were predicted, and functional enrichment results showed that the enriched genes were involved in IR- and glycolipid metabolism-related processes. Additionally, the coexpression network was also constructed to further reflect the potential functions of the DE lncRNAs.

**Conclusion:**

The study describes the expression profiles of lncRNAs and mRNAs and the functional networks involved in HFD-induced hepatic IR. These findings may provide a new perspective for the study of lncRNAs in hepatic IR- and glycolipid metabolism-related diseases.

## Introduction

Insulin resistance (IR) is considered to be associated with many metabolic disorders, such as type 2 diabetes and obesity. The liver is a main target organ for the action of insulin in the body. Under normal physiological conditions, insulin promotes anabolic metabolism in the liver by enhancing glucose consumption and lipid synthesis; however, the IR state is characterized by a failure to inhibit hepatic glucose production, with paradoxically increased fat accumulation, resulting in hyperglycemia and hypertriglyceridemia [1, 2]. Therefore, elucidating the pathogenic mechanism underlying hepatic IR is key to preventing and treating metabolic disorders and related diseases.

Long noncoding RNAs (lncRNAs) belong to a class of regulatory noncoding RNAs with transcript lengths greater than 200 nucleotides and serve vital roles in transcriptional and posttranscriptional gene expression regulation [3, 4]. Recently, dysregulation of lncRNAs has been found to be important in various human diseases, such as cancer, diabetes and cardiovascular diseases [5–7]. In particular, some reports have noted that lncRNAs play an important role in liver glycolipid metabolism. For instance, Zhu et al. showed that the expression of the lncRNA MEG3 was increased in high-fat diet (HFD)-fed and ob/ob mice and upregulated by palmitate, oleate or linoleate. The suppression of the lncRNA MEG3 improved the elevation in triglyceride levels and the impaired glucose tolerance and downregulation of the glucogen content in the HFD-fed mice or ob/ob mice [8]. However, lncRNAs specifically involved in the progression of hepatic IR are poorly characterized.

In our previous studies, hepatic IR in mice was established with HFD feeding [9]. In an attempt to understand the mechanisms underlying the lncRNAs involved in hepatic IR, in this study, systematic analyses of the hepatic expression profiles of lncRNAs and mRNAs in normal diet (ND)-fed mice and HFD-induced hepatic IR mice were performed. DE lncRNAs and mRNAs were identified in these two groups of mice. In addition, some of the DE lncRNAs were verified by quantitative real-time PCR (q-PCR). The target genes of the DE lncRNAs were also predicted, and biological functions and pathway enrichment were analyzed by Gene Ontology (GO) and Kyoto Encyclopedia of Genes and Genomes (KEGG) enrichment analyses. Moreover, interaction and coexpression networks were constructed for the DE mRNAs and lncRNAs to determine the regulatory roles of lncRNAs in hepatic IR.

## Materials and methods

### Animal experiments

Ten liver tissue samples were derived from HFD-induced liver IR mice and ND-fed mice in our previous study (ND, *n* = 5; HFD, *n* = 5) [9]. Briefly, male 8-week-old C57BL/6 J mice were purchased from Vital River Laboratory Animal Technology (Beijing, China). After 1 week of adaptive feeding, mice were randomly assigned to either a ND (*n* = 12) group or a HFD (*n* = 12) group, and were fed for 12 weeks. Diet formula was showed in our previous study [9]. At the end of the 12th week of the feeding experiment, five mice randomly selected from each group were sacrificed for liver tissue collection.

### RNA extraction, library construction, and high-throughput sequencing

High-throughput sequencing was performed on the liver tissue samples of each mouse individually (Majorbio Bio-Pham Technology Co., Shanghai, China). Briefly, total RNA was extracted from the liver tissue using TRIzol® Reagent (Invitrogen, Carlsbad, CA, USA), and the concentration and purity of the extracted RNA were detected by using a NanoDrop 2000 spectrophotometer (Thermo Scientific, Wilmington, DE, USA). The integrity of the isolated RNA was evaluated by agarose gel electrophoresis, and the RNA integrity number was determined with an Agilent 2100 (Agilent Technologies, Palo Alto, CA, USA). Total RNA without rRNA was utilized for RNA sample preparation using a Ribo-Zero Magnetic kit (EpiCentre Biotechnologies, Madison, WI, USA). Subsequently, RNA libraries were prepared by using the rRNA-depleted RNA with the TruSeq™ Stranded Total RNA Library Prep Kit (Illumina, San Diego, CA, USA). High-throughput sequencing was performed using the HiSeq 4000 SBS Kit (Illumina, San Diego, CA, USA).

### Analysis of sequencing data

Through quality control of the raw reads produced by high-throughput sequencing, clean reads were obtained. The raw reads were processed by removing the reads with no insert clip, an N ratio exceeding 10%, sequences < 20 nucleotides (nt) and low quality. The Q20 and Q30 content of the clean data were subsequently calculated. The high-quality reads were used to analyze lncRNAs and mRNAs by mapping to a mouse reference genome with HISAT2 software [10]. The remaining reads without comment information were analyzed by StringTie novel transcript [11].

### Identification of lncRNAs

Known lncRNAs were identified by comparing the transcripts of the existing reference genome and reported lncRNA sequences from a collection of lncRNA-related databases including NONCODE, Ensembl, NCBI, UCSC, LncRNAdb, GENCODE, and LncRNADisease. Novel lncRNAs were identified by filtering step by step. Preliminary filtering included removing mRNAs and selecting the transcripts with a length ≥ 200 bp, exon number ≥ 2, ORF length ≤ 300 bp and fragment count ≥3 by using gffcompare information to filter different types of lncRNAs (intergenic lncRNAs, intronic lncRNAs, and antisense lncRNAs). Advanced filters of the coding potential of lncRNAs included a Coding Potential Calculator (CPC) score < 0, a Coding-Non-Coding Index (CNCI) score < 0, a Coding Potential Assessment Tool (CPAT) result indicating low coding potential and no significance in the Pfam database.

### Expression analysis of lncRNAs and mRNAs

RSEM can be used to calculate the expression of genes or transcripts from single- or double-terminal sequencing data. The quantitative expression of both lncRNAs and mRNAs in each sample was calculated in fragments per kilo-base of exon per million reads mapped. Transcripts with a *P* value < 0.05 and |log2 (HFD/ND)| ≥ 1 were determined to be DE genes and transcripts. A Volcano plot and hierarchical clustering were used to analyze the DE lncRNAs and mRNAs identified between ND- and HFD-fed mice.

### Q-PCR

The expression of seven lncRNAs in the liver of ten mice (ND, *n* = 5; HFD, *n* = 5) was assessed using q-PCR to verify the accuracy of the high-throughput sequencing results. Total RNA was extracted from samples by using TRIzol® Reagent (Invitrogen, Carlsbad, CA, USA), and cDNA was synthesized by using the miScript II RT Kit (Qiagen, Hilden, Germany). Real-time PCR was performed with the miScript SYBR® Green PCR Kit (Qiagen, Hilden, Germany) using a 7500 FAST real-time PCR system (Applied Biosystems, Foster City, CA, USA). The relative expression of lncRNAs was calculated by the 2^-△△Ct^ method. A two-tailed Student’s *t* test was used to compare lncRNA expression between samples from the ND group and those from the HFD group in 3 experimental replicates. The forward and reverse primers for lncRNAs are shown in supplemental file 1. The PCR products were sequenced to verify that they had been identified correctly.

### GO and KEGG enrichment analyses

The prediction of lncRNA functions is based on the functional annotations of related *cis-* and *trans-*target genes. The protein-coding genes located within 100 kb upstream or downstream of the identified DE lncRNAs were defined as *cis*-target genes. The RNAplex program was used to identify possible *trans*-target genes of the DE lncRNAs. Then, GO and KEGG enrichment analyses were performed to predict the biological functions of the DE lncRNA target genes. GO analysis was applied for the identification and annotation of genes in the three categories of molecular functions, biological processes, and cellular components. KEGG enrichment analysis was used to analyze the important pathways involving the target genes of the DE lncRNAs. The threshold (a *P* value ≤0.05 and false discovery rate ≤ 0.05) was calculated using Fisher’s exact test.

### Interaction analysis and coexpression network analysis

Interactions among the identified DE mRNAs were evaluated with Search Tool for the Retrieval of Interacting Genes/Proteins (STRING). These genes required an interaction score ≥ 0.4, and the interaction network was built with Cytoscape (version 3.6.1).

A weighted gene coexpression network (WGCNA) can be used to find clusters (modules) of highly correlated genes. The coexpression network of the DE lncRNAs and mRNAs was constructed by using the R package WGCNA. We used step-by-step network construction with a soft threshold of β = 16 (R^2^ = 0.43) and a minimum module size of 30. The topological overlap distance calculated from the adjacency matrix was then clustered with average linkage hierarchical clustering. The connections in each module were visualized using Cytoscape (version 3.6.1).

## Results

### Sequencing data summary

In our study, libraries were constructed from liver tissue samples from ND-fed mice (*n* = 5) and HFD-induced hepatic IR mice (*n* = 5) and subjected to sequencing analysis. High-throughput sequencing produced 516,429,450 and 457,341,558 raw reads from the two groups, respectively. The raw reads were filtered to obtain high-quality clean reads. In total, 506,933,232 and 448,761,362 clean reads were retained for the ND and HFD groups, respectively (Table 1). The Q20 and Q30 of the clean data were subsequently calculated to evaluate the quality of the RNA-sequencing data. The Q20 and Q30 quality scores were higher than 90%, and the sequencing error rate was less than 1%. The data indicated that the RNA-sequencing results had reliable quality. The obtained clean reads were mapped to a mouse reference genome, with the mapping ratio ranging from 96.10 to 97.13% (supplemental file 2).

**Table 1:** Results of sequencing before and after quality control of ND and HFD libraries

	ND		HFD	
	Raw data	Clean data	Raw data	Clean data
Total_Reads	516,429,450	506,933,232	457,341,558	448,761,362
Total_Bases	77,980,846,950	68,519,999,495	69,058,575,258	60,357,317,965
Error%	0.01242	0.0112	0.0124	0.01116
Q20%	97.762	98.806	97.748	98.81
Q30%	94.532	96.266	94.516	96.274

Error% means base Error rate. Q20% and Q30% means the percentage of bases with Phred value greater than 20 and 30 in the total bases. ND, normal diet mice; HFD, high-fat diet mice

### Identification and classification of lncRNAs in the liver of mice

**Fig. 1:**
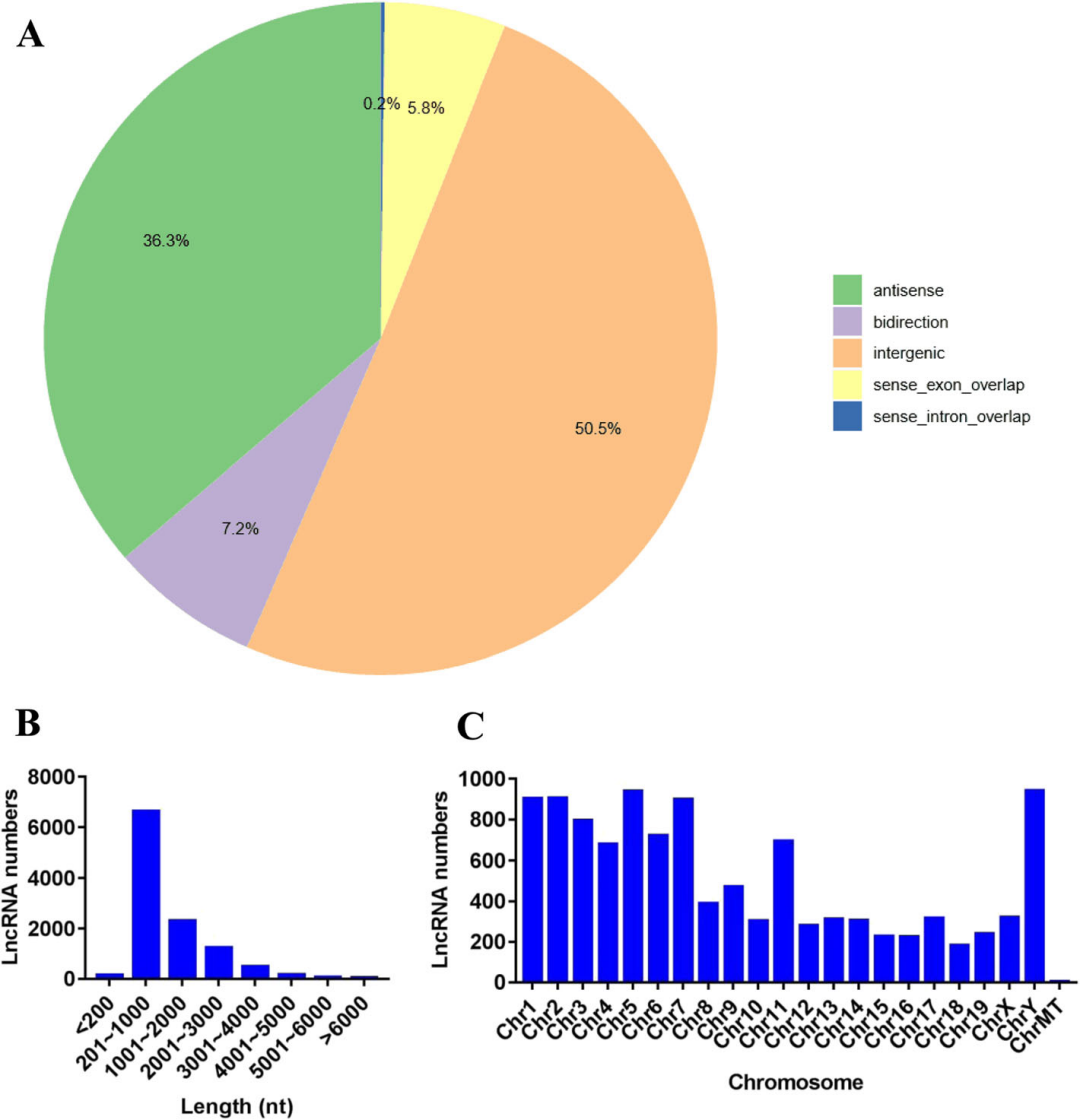
Expression characteristics of lncRNAs. **a** Classification of lncRNAs. **b** Length distribution of lncRNAs. **c** Chromosomal distribution of lncRNA numbers

Based on the mouse reference genome and related databases, 10,495 known lncRNAs were found. After a strict filtering process, 541 novel lncRNAs, which exhibited repeated emergence in four software programs (CPC, PfamScan, CPAT and CNCI), were identified (supplemental file 3). According to the relative chromosomal position of the coding gene, the novel lncRNAs were classified into five broad categories: 5572 intergenic lncRNAs (50.5%), 4004 antisense lncRNAs (36.3%), 642 sense exonic overlap lncRNAs (5.8%), 22 sense intronic overlap lncRNAs (0.2%) and 796 bidirectional lncRNAs (7.2%) (Fig. 1a). Furthermore, the distributions of these lncRNAs in terms of length and chromosomal location were analyzed in this study. In different length groups, most of the lncRNAs were 201–1000 nt long (Fig. 1b). Chromosomal distribution analysis revealed that chromosomes 1, 2, 5 and Y contained relatively higher amounts of lncRNAs (Fig. 1c).

### Differential expression analysis of lncRNAs and mRNAs

**Fig. 2:**
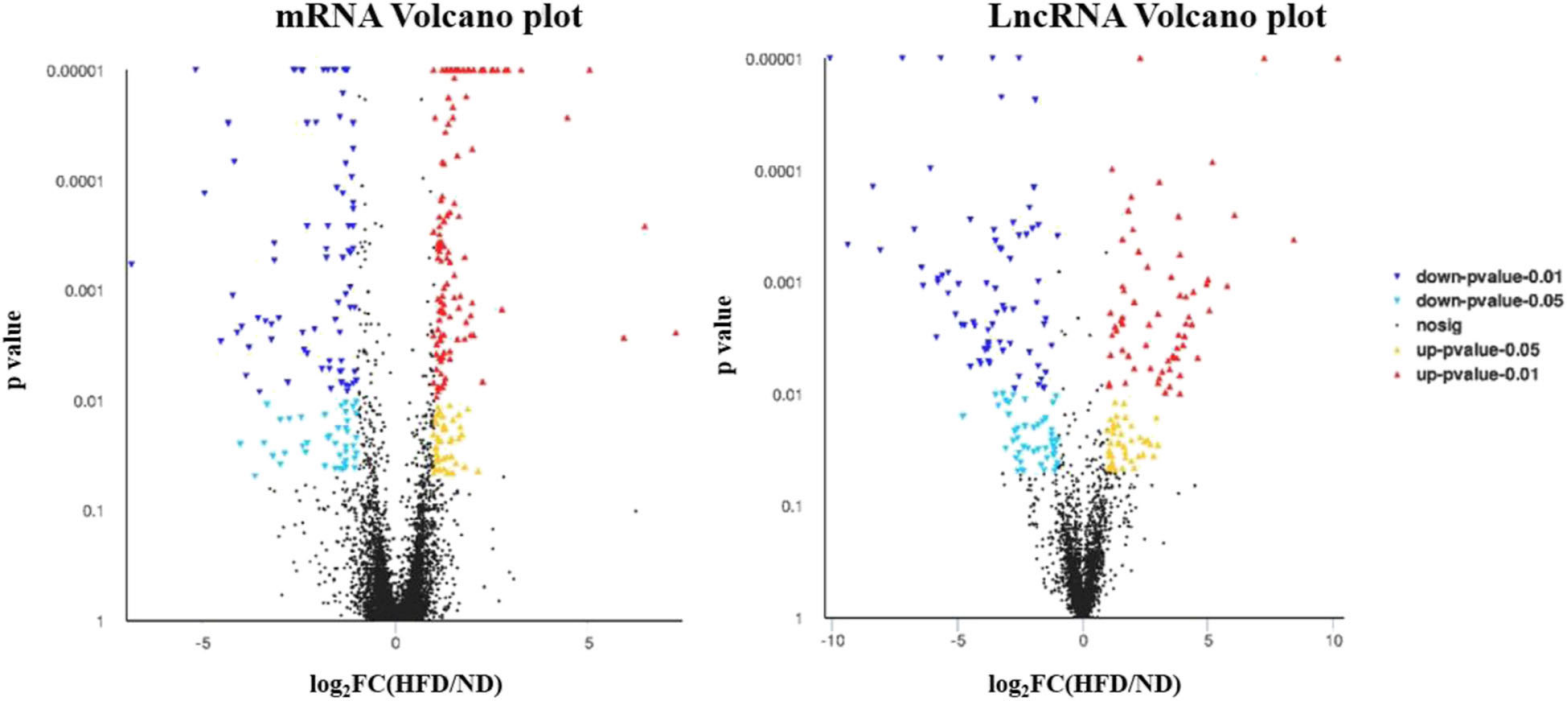
Volcano plots of mRNAs (left) and lncRNAs (right) differentially expressed between the two groups. Red and yellow spots represent upregulated mRNAs or lncRNAs, and blue spots indicate downregulated mRNAs or lncRNAs. Black spots represent mRNAs or lncRNAs that did not show obvious changes between the ND and HFD groups. ND, normal diet-fed mice; HFD, high-fat diet-fed mice

**Fig. 3:**
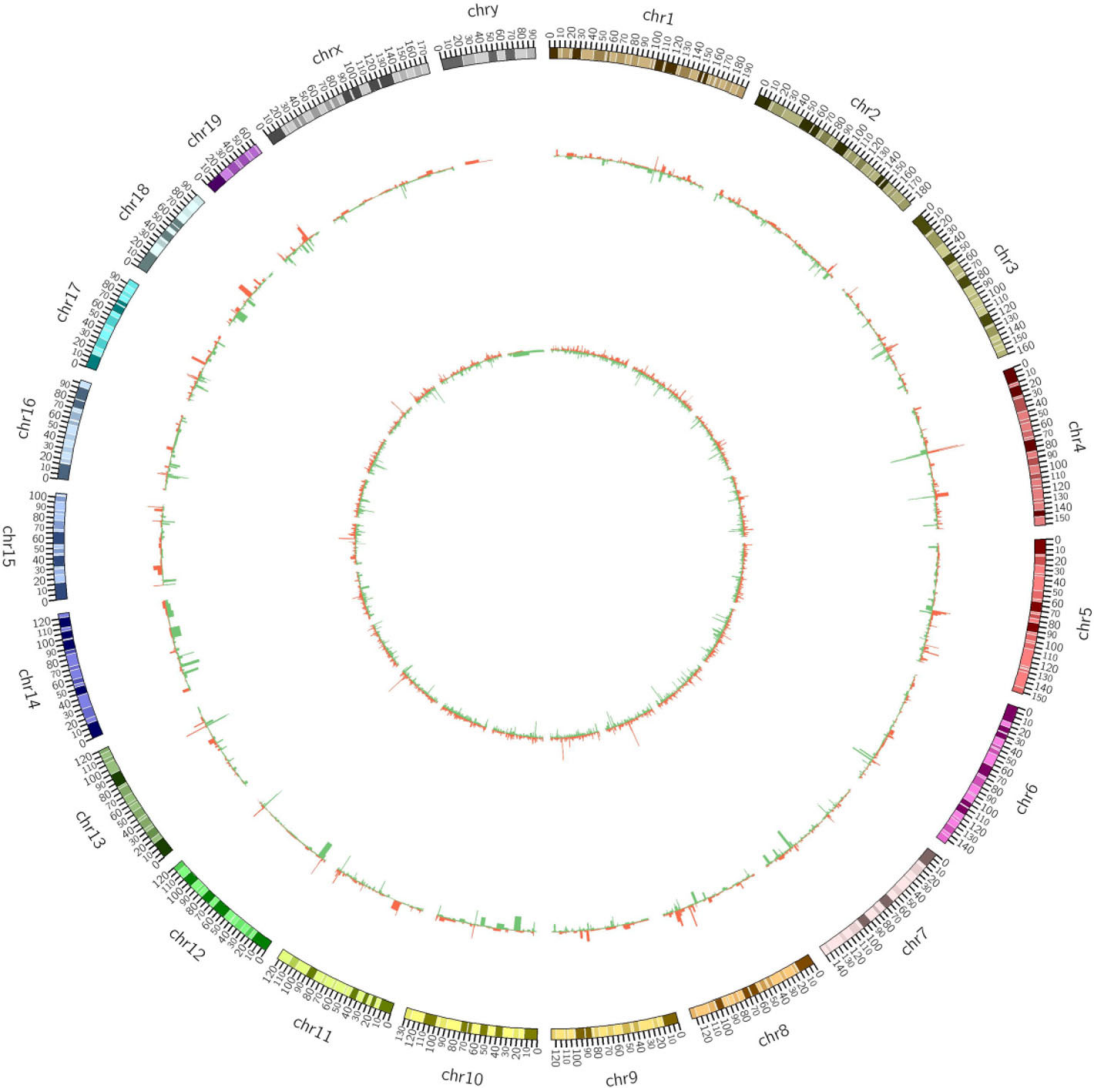
Distributions of differentially expressed lncRNAs and mRNAs on chromosomes. The outer layer shows the distribution on chromosomes, the middle layer indicates the differential expression of lncRNAs on the chromosomes, and the inner layer shows the distribution of differentially expressed mRNAs on the chromosomes. Red indicates upregulation, green indicates downregulation, and the height indicates the fold-change value

After high-throughput sequencing, a total of 11,036 lncRNAs were obtained by comparing the ND and HFD groups. The analysis of the lncRNAs DE between the two groups showed that compared with the ND group, the HFD group contained 232 DE lncRNAs, among which 108 were upregulated lncRNAs and 124 were downregulated lncRNAs. Regarding the expression profiles of mRNAs, 40,675 mRNAs were present in both groups. Among all of these mRNAs, compared with the ND group, the HFD group contained 291 DE mRNAs, including 166 upregulated mRNAs and 125 downregulated mRNAs. A volcano plot and heatmap were generated to visualize the DE lncRNAs and DE mRNAs identified by comparing the two groups, as shown in Fig. 2 and in supplemental file 4. Moreover, the chromosomal distributions of the DE lncRNAs and mRNAs are shown in Fig. 3.

### DE lncRNA validation by q-PCR

**Fig. 4:**
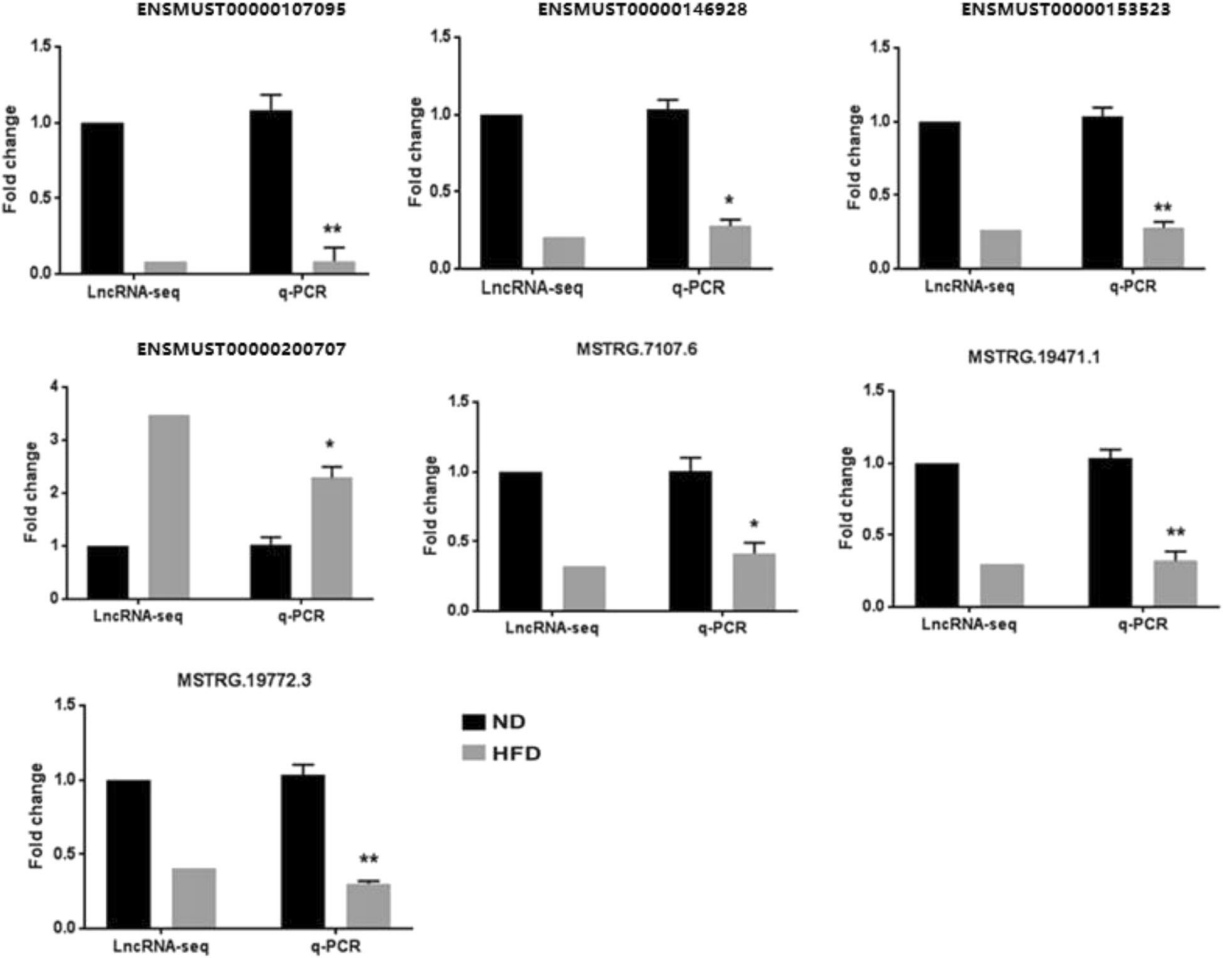
q-PCR validation of seven differentially expressed lncRNAs identified using high-throughput sequencing of mouse liver tissue samples. ND, normal diet-fed mice; HFD, high-fat diet-fed mice. *P < 0.05 and **P < 0.01 compared with the value for the ND group

To validate the reliability of the lncRNA expression data, seven lncRNAs that exhibited relatively abundant expression and significant differential expression in the livers from mice in the two groups were selected and evaluated by q-PCR. These lncRNAs included one upregulated lncRNA (ENSMUST00000200707) and six downregulated lncRNAs (ENSMUST00000107095, ENSMUST00000146928, ENSMUST00000153523, MSTRG.7107.6, MSTRG.19471.1 and MSTRG.19772.3). The results showed that the level of the lncRNA ENSMUST000030200707 was significantly increased, while the levels of the lncRNAs ENSMUST00000107095, ENSMUST00000146928, ENSMUST00000153523, MSTRG.7107.6, MSTRG.19471.1 and MSTRG.19772.3 were obviously decreased in the HFD group compared with the ND group (Fig. 4). The q-PCR validation results for these DE lncRNAs were consistent with our bioinformatic analysis based on high-throughput sequencing.

### GO and KEGG enrichment analyses of the DE lncRNAs and mRNAs

**Fig. 5:**
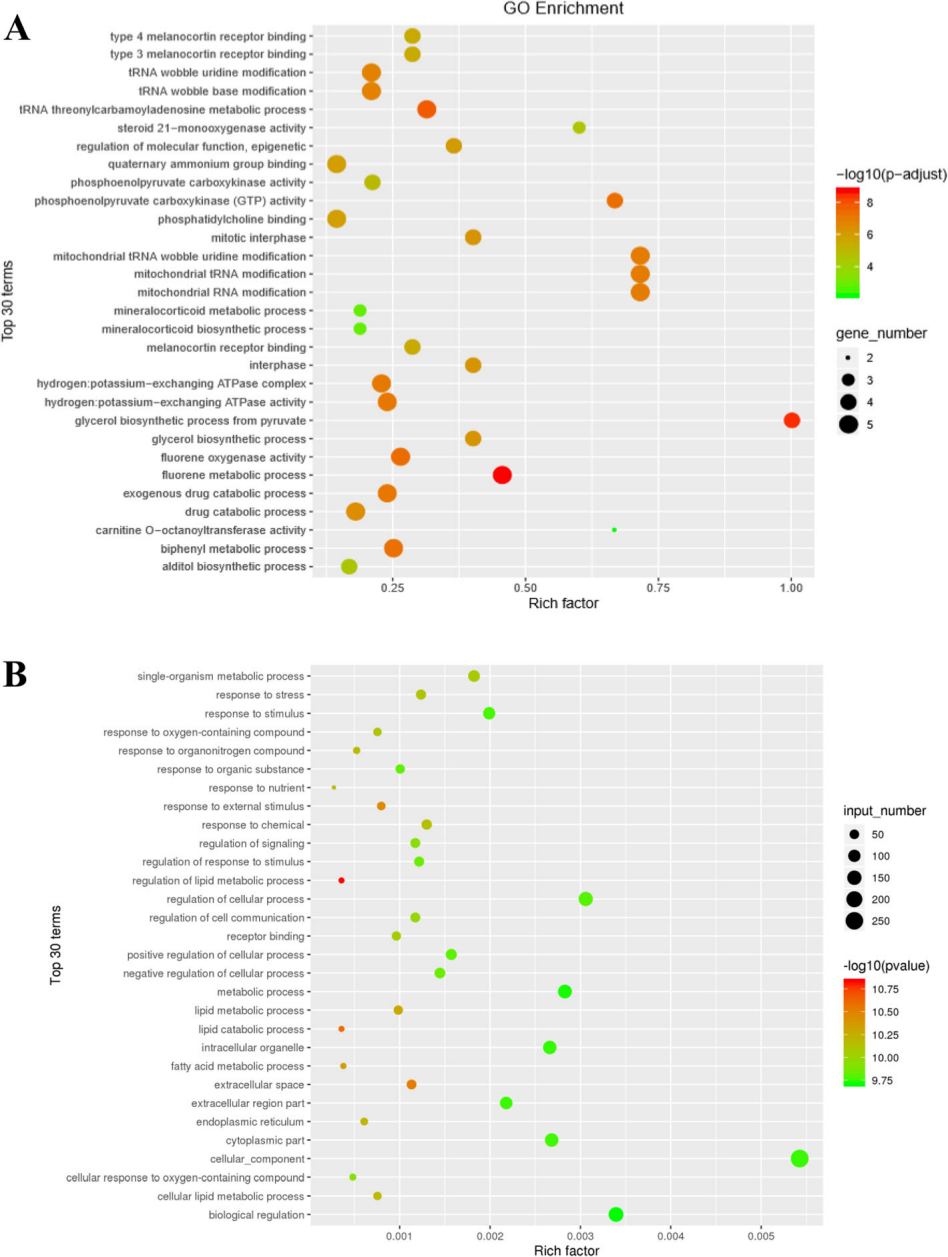
The top 30 GO terms for differentially expressed genes. **a** The top 30 GO terms for the target genes of differentially expressed lncRNAs. **b** The top 30 GO terms for differentially expressed mRNAs. The circle size represents the gene number. The Q value is indicated by the color gradient

**Fig. 6:**
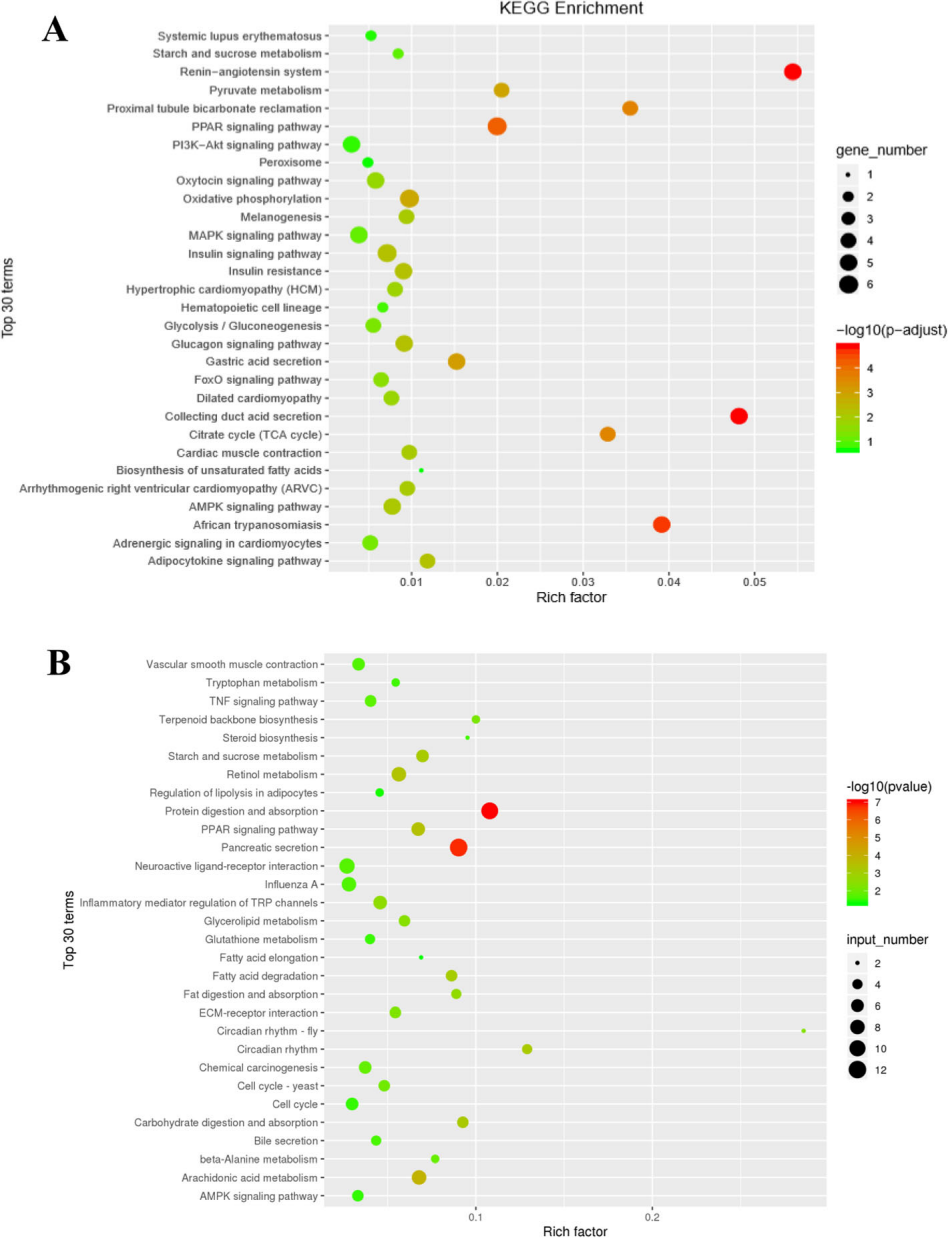
The top 30 KEGG pathways for differentially expressed genes. **a** The top 30 KEGG pathways for the target genes of differentially expressed lncRNAs. **b** The top 30 KEGG pathways for differentially expressed mRNAs. The circle size represents the gene number. The Q value is indicated by the color gradient

First, target genes of the DE lncRNAs were predicted by bioinformatic approaches, and the results are presented in supplemental file 5. Subsequently, GO and KEGG enrichment analyses were performed on the predicted target genes of the DE lncRNAs. The top 30 GO terms are shown in Fig. 5a, in which “glycerol biosynthetic process from pyruvate” was majorly enriched in the biological process category. GO enrichment also indicated enrichment of other important biological processes, including various compounds and nucleic acid metabolism in the cell, mitochondrial modification, catalytic enzyme activity and molecular function regulation. Furthermore, the top 30 enriched KEGG pathways are presented in Fig. 6a, and the “Renin-angiotensin system” was the top enriched pathway. More importantly, pathway enrichment analysis showed enrichment of several pathways related to IR or glycolipid metabolism, such as the citrate cycle (TCA cycle), the PPAR signaling pathway, the AMPK signaling pathway, the MAPK signaling pathway, glycolysis/gluconeogenesis, the glucagon signaling pathway, biosynthesis of unsaturated fatty acids, the PI3K-Akt signaling pathway, insulin resistance, and the insulin signaling pathway.

For the DE mRNAs, the top 30 enriched GO terms and KEGG pathways are shown in Fig. 5b and Fig. 6b. The GO terms mainly included cellular_component, biological regulation, regulation of cellular process, metabolic process, intracellular organelle and cytoplasmic part. KEGG pathway analysis showed that DE mRNAs were mainly associated with Protein digestion and absorption, Pancreatic secretion, Arachidonic acid metabolism, PPAR signaling pathway and Retinol metabolism.

### Interaction and coexpression network analysis

**Fig. 7:**
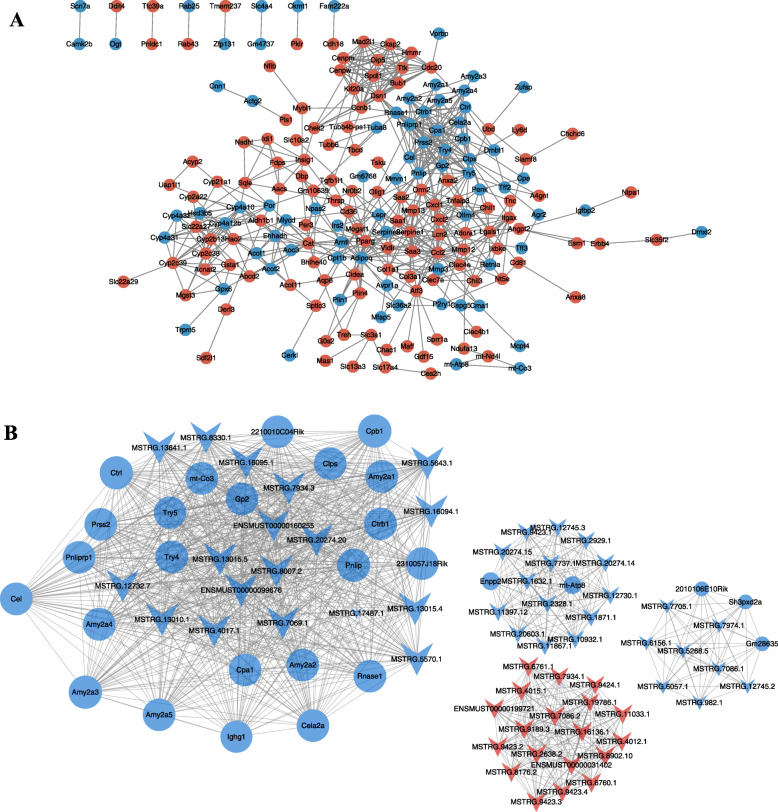
Interaction and coexpression network analyses. **a** Interactions between differentially expressed mRNAs. The red circle represents upregulated mRNAs, and the blue circle represents downregulated mRNAs. **b** Coexpression network of differentially expressed lncRNAs and mRNAs. The red arrow represents upregulated lncRNAs, the blue arrow represents downregulated lncRNAs, the blue circle represents downregulated mRNAs, the grey line represents correlation of genes, and node size represents the number of linked lines

The interactions among the proteins encoded by the DE mRNAs are shown in Fig. 7a. The Cxcl2, Serpine1 and Prss2 genes were the important genes that interacted with many of the other DE mRNAs in this network. Moreover, a DE lncRNA-mRNA coexpression network, which consisted of 87 nodes (59 DE lncRNAs and 28 DE mRNAs) and 1125 edges, was constructed (Fig. 7b). Within this RNA network, the lncRNA MSTRG.8007.2 had the maximum number of targets. The Pnlip, Ctrl, Cpb1 and Amy2a3 genes had the maximum number of coexpressed lncRNAs.

## Discussion

Increasing evidence has shown that the abnormal expression of lncRNAs is closely related to the occurrence and development of IR and related diseases [8, 12, 13]. Nevertheless, a comprehensive database of the potential role of lncRNAs in the pathogenesis of hepatic IR still lacks. Therefore, in this study, using high-throughput RNA sequencing, we obtained the expression profiles of lncRNA and mRNA in the liver of ND-fed mice and HFD-induced hepatic IR mice. On this basis, we analyzed DE lncRNAs and mRNAs between the two groups and explored the potential roles of lncRNAs in progress of HFD-induced hepatic IR by various bioinformatics methods. Our results provided the valuable information for the understanding of the lncRNA function in HFD-induced hepatic IR.

In this study, after high-throughput sequencing, a total of 11,036 lncRNAs were obtained in livers of the two groups of mice. Among these lncRNAs, most of the transcripts were intergenic lncRNAs. DE lncRNA analysis showed that compared with ND mice, HFD-induced hepatic IR mice expressed 232 DE lncRNAs in the liver, including 108 upregulated lncRNAs and 124 downregulated lncRNAs. Subsequently, q-PCR was performed to validate the high-throughput sequencing results for the DE lncRNAs. Seven DE lncRNAs, which were relatively enriched in the liver and significantly DE, were subjected to experimental verification. The results showed that the expression changes in the seven lncRNAs detected by q-PCR were consistent with the sequencing results, indicating the reliability of the lncRNA sequencing data. In addition, by examining the literature, we found that the lncRNA ENSMUST00000107095 (lncRNA Gm15441), a DE lncRNA validated by q-PCR in our study, has been recently reported to be a regulator of fatty acid oxidation in hepatocytes [14]. This result further confirms the reliability and reflects the application value of our results, which provide a basis for further study of the mechanism involving lncRNAs in hepatic IR.

For the expression profiles of mRNAs, 40,675 mRNAs were present in both groups. Compared with the ND group, the HFD group exhibited 166 mRNAs with significantly upregulated expression and 125 mRNAs were significantly downregulated expression. Some of these DE mRNAs, such as Irs2 [15, 16] and Pparg [17, 18], have been shown to be closely related to the development of IR or the process of glycolipid metabolism. Moreover, the results for interactions among the DE mRNAs showed that some mRNAs, such as Cxcl2 and Serpine1, interacted with many other DE mRNAs, suggesting that they may be crucial in pathological changes that occur during HFD-induced hepatic IR and glycolipid metabolism disorder. In addition, this hypothesis has also been tested in some studies. For instance, PAI-1, which is encoded by the Serpinel gene, has been reported to exhibit elevated expression in obesity and type 2 diabetes [19, 20]. Coudriet GM et al. recently showed that the absence of PAI-1 resulted in improvements in glucose tolerance and IR [21].

The prediction of lncRNA functions is based on the functional annotation of lncRNA target genes. Thus, target genes of the DE lncRNAs were identified, and GO and KEGG enrichment analyses were applied to determine the functional distributions of these target genes. The GO functional enrichment analysis showed that the target genes associated with the DE lncRNAs were significantly involved in glycerol biosynthesis from pyruvate and various compounds and nucleic acid metabolism in the cell. It is worth noting that the target genes were significantly enriched in a variety of IR- and glycolipid metabolism-related pathways, such as the citrate cycle (TCA cycle), PPAR signaling pathway, PI3K-Akt signaling pathway and insulin signaling pathway, by the KEGG enrichment analysis. Therefore, the finding effectively suggests that these DE lncRNAs identified in HFD-induced hepatic IR mice may be involved in mediating the process of IR by regulating various signaling pathways.

Coexpression network analysis of DE lncRNAs and mRNAs is helpful for further investigating the potential functions of lncRNAs. In the network we constructed, most of the mRNAs coexpressed with the lncRNAs encoded digestive or metabolic enzymes. Some of these mRNAs have been reported to be associated with IR. For example, Nishimura et al. found that Enpp2 contributed to adipose tissue expansion and IR in diet-induced obesity [22]. Reeves et al. also reported that there is a relatively strong and consistent relationship between serum Enpp2 and glucose homeostasis or insulin sensitivity in relatively old, nondiabetic humans who were overweight or obese [23]. Thus, whether those lncRNAs coexpressed with Enpp2 are also involved in the process of IR needs to be further verified by experiments.

Furthermore, there were some limitations to this study. First, in theory, the expression levels of each DE lncRNAs in HFD-induced hepatic IR need to be validated using q-PCR, while our study only verified 7 DE lncRNAs. Thus, all DE lncRNAs in our study may only be regarded as a lncRNA reference dataset, and more further research needed to be carried out to validate the expression of lncRNAs of interest. Second, though the potential functions of the DE lncRNAs were predicted through GO and KEGG enrichment analyses of their target genes and coexpression network analysis of DE lncRNAs and mRNAs, these results lack experimental validation. Therefore, the specific lncRNAs that function in hepatic IR and their associated mechanisms should be further verified by in vivo and in vitro study in the future.

## Conclusions

In summary, our study systematically analyzed the expression profiles of lncRNAs and mRNAs in the liver of ND-fed mice and HFD-induced hepatic IR mice, and identified DE lncRNAs and mRNAs between the two groups. The results of relevant biological functions and pathway enrichment analysis indicate that these DE lncRNAs may be involved in glycolipid metabolism and mediating the process of IR by regulating various signaling pathways. Our results provided not only a systematic perspective on lncRNAs in hepatic IR but also a valuable data resource for further study of the potential functions of lncRNAs in the pathogenesis of hepatic IR and glycolipid metabolism-related diseases.

## Supplementary information

**Additional file 1.**

**Additional file 2.**

**Additional file 3.**

**Additional file 4.**

**Additional file 5.**

## Data Availability

All data generated or analyzed during this study are included in this published article (and its supplementary information files).

## Abbreviations

IR: Insulin resistance
lncRNAs: Long noncoding RNAs
ND: Normal diet
HFD: High-fat diet
DE: Differentially expressed
q-PCR: Quantitative real-time PCR
GO: Gene Ontology
KEGG: Kyoto Encyclopedia of Genes and Genomes
CPC: Coding Potential Calculator
CNCI: Coding-Non-Coding Index
CPAT: Coding Potential Assessment Tool

## References

[1] Santoleri D, Titchenell PM (2019). Resolving the paradox of hepatic insulin resistance. Cell Mol Gastroenterol Hepatol.

[2] Méndez-Sánchez N, Chávez-Tapia NC, Zamora-Valdés D, Medina-Santillán R, Uribe M (2007). Hepatobiliary diseases and insulin resistance. Curr Med Chem.

[3] Coe FL, Evan A, Worcester E (2005). Kidney stone disease. J Clin Invest.

[4] Polovic M, Dittmar S, Hennemeier I, Humpf H-U, Seliger B, Fornara P (2018). Identification of a novel lncRNA induced by the nephrotoxin ochratoxin a and expressed in human renal tumor tissue. Cell Mol Life Sci.

[5] Kopp F, Mendell JT (2018). Functional classification and experimental dissection of long noncoding RNAs. Cell.

[6] Uchida S, Dimmeler S (2015). Long noncoding RNAs in cardiovascular diseases. Circ Res.

[7] Huarte M (2015). The emerging role of lncRNAs in cancer. Nat Med.

[8] Zhu X, Wu Y-B, Zhou J, Kang D-M (2016). Upregulation of lncRNA MEG3 promotes hepatic insulin resistance via increasing FoxO1 expression. Biochem Biophys Res Commun.

[9] Zhao X, Chen Z, Zhou Z, Li Y, Wang Y, Zhou Z (2019). High-throughput sequencing of small RNAs and analysis of differentially expressed microRNAs associated with high-fat diet-induced hepatic insulin resistance in mice. Genes Nutr.

[10] Kim D, Langmead B, Salzberg SL (2015). HISAT: a fast spliced aligner with low memory requirements. Nat Methods.

[11] Trapnell C, Roberts A, Goff L, Pertea G, Kim D, Kelley DR (2012). Differential gene and transcript expression analysis of RNA-seq experiments with TopHat and cufflinks. Nat Protoc.

[12] Yan C, Chen J, Chen N (2016). Long noncoding RNA MALAT1 promotes hepatic steatosis and insulin resistance by increasing nuclear SREBP-1c protein stability. Sci Rep.

[13] Goyal N, Kesharwani D, Datta M (2018). Lnc-ing non-coding RNAs with metabolism and diabetes: roles of lncRNAs. Cell Mol Life Sci.

[14] Batista TM, Garcia-Martin R, Cai W, Konishi M, O'Neill BT, Sakaguchi M (2019). Multi-dimensional transcriptional remodeling by physiological insulin in vivo. Cell Rep.

[15] Dong X, Park S, Lin X, Copps K, Yi X, White MF (2006). Irs1 and Irs2 signaling is essential for hepatic glucose homeostasis and systemic growth. J Clin Invest.

[16] Honma M, Sawada S, Ueno Y, Murakami K, Yamada T, Gao J (2018). Selective insulin resistance with differential expressions of IRS-1 and IRS-2 in human NAFLD livers. Int J Obes.

[17] Vidal-Puig A, Jimenez-Liñan M, Lowell BB, Hamann A, Hu E, Spiegelman B (1996). Regulation of PPAR gamma gene expression by nutrition and obesity in rodents. J Clin Invest.

[18] Zhang Y-L, Hernandez-Ono A, Siri P, Weisberg S, Conlon D, Graham MJ (2006). Aberrant hepatic expression of PPARgamma2 stimulates hepatic lipogenesis in a mouse model of obesity, insulin resistance, dyslipidemia, and hepatic steatosis. J Biol Chem.

[19] Shimomura I, Funahashi T, Takahashi M, Maeda K, Kotani K, Nakamura T (1996). Enhanced expression of PAI-1 in visceral fat: possible contributor to vascular disease in obesity. Nat Med.

[20] Juhan-Vague I, Alessi MC (1997). PAI-1, obesity, insulin resistance and risk of cardiovascular events. Thromb Haemost.

[21] Coudriet GM, Stoops J, Orr AV, Bhushan B, Koral K, Lee S (2019). A noncanonical role for plasminogen activator inhibitor type 1 in obesity-induced diabetes. Am J Pathol.

[22] Nishimura S, Nagasaki M, Okudaira S, Aoki J, Ohmori T, Ohkawa R (2014). ENPP2 contributes to adipose tissue expansion and insulin resistance in diet-induced obesity. Diabetes.

[23] Reeves VL, Trybula JS, Wills RC, Goodpaster BH, Dubé JJ, Kienesberger PC (2015). Serum Autotaxin/ENPP2 correlates with insulin resistance in older humans with obesity. Obesity (Silver Spring, Md).

