# Supplementary material for: Integrated analysis of differentially expressed long noncoding RNAs and mRNAs associated with high-fat diet-induced hepatic insulin resistance in mice
Source: Nutr Metab (Lond). 2020 Jun 18;17:45. doi: 10.1186/s12986-020-00467-7 (PMC7302146; doi:10.1186/s12986-020-00467-7)
Supplement: Supplementary file 3 — Additional file 3. [file 12986_2020_467_MOESM3_ESM.docx]

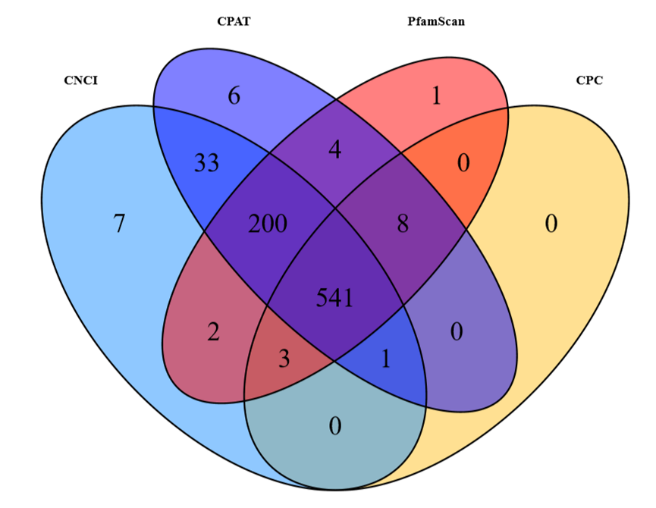


**Supplemental file 3. Venn Diagram of screening novel lncRNA using four** **coding potential prediction software.** Blue represents CNCI software, purple represents CPAT software, red represents PfamScan software, and yellow represents CPC software.
