# Supplementary material for: Integrated analysis of differentially expressed long noncoding RNAs and mRNAs associated with high-fat diet-induced hepatic insulin resistance in mice
Source: Nutr Metab (Lond). 2020 Jun 18;17:45. doi: 10.1186/s12986-020-00467-7 (PMC7302146; doi:10.1186/s12986-020-00467-7)
Supplement: Supplementary file 4 — Additional file 4. [file 12986_2020_467_MOESM4_ESM.pdf]

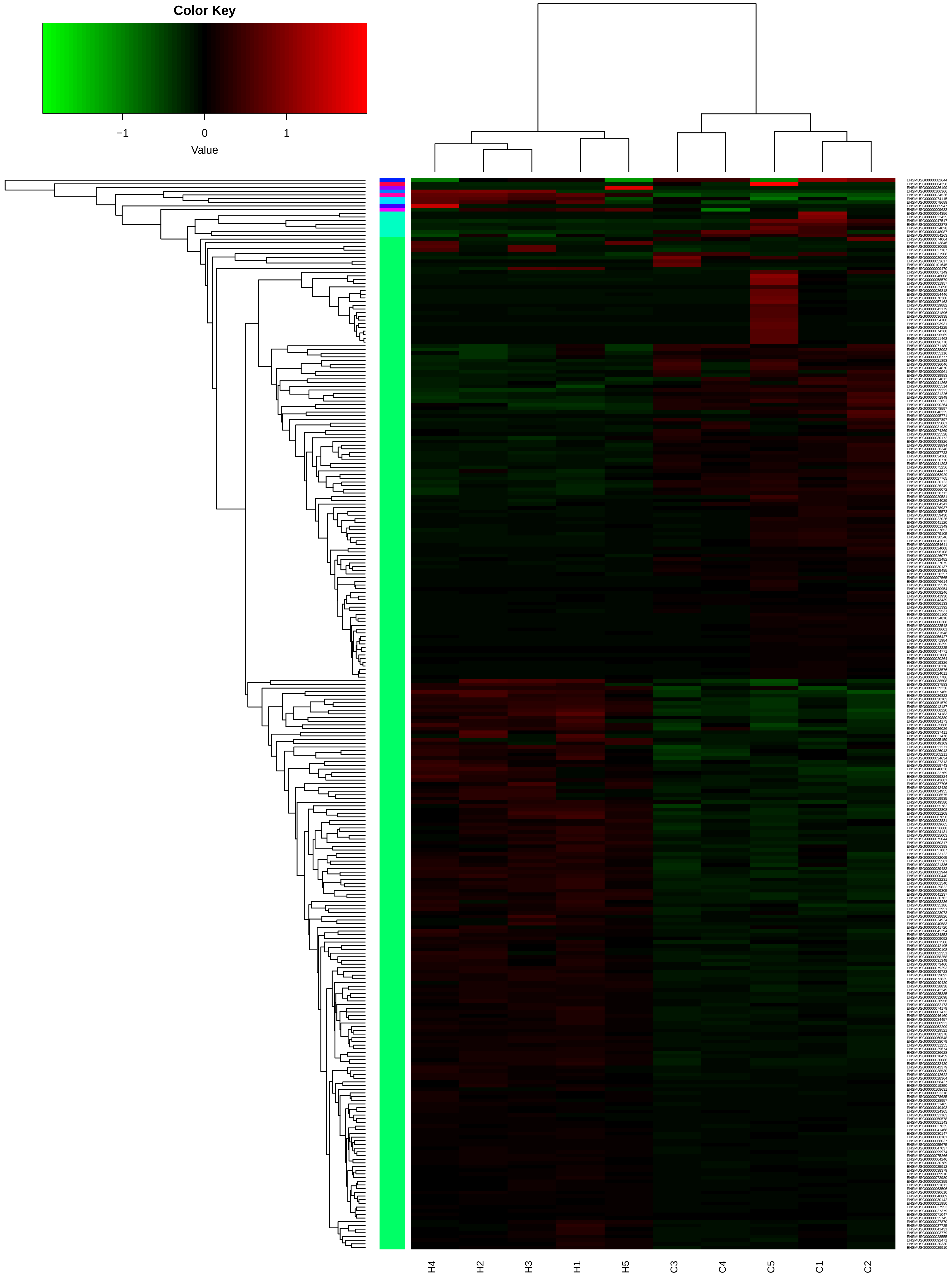

Heatmap of differentially expressed mRNAs

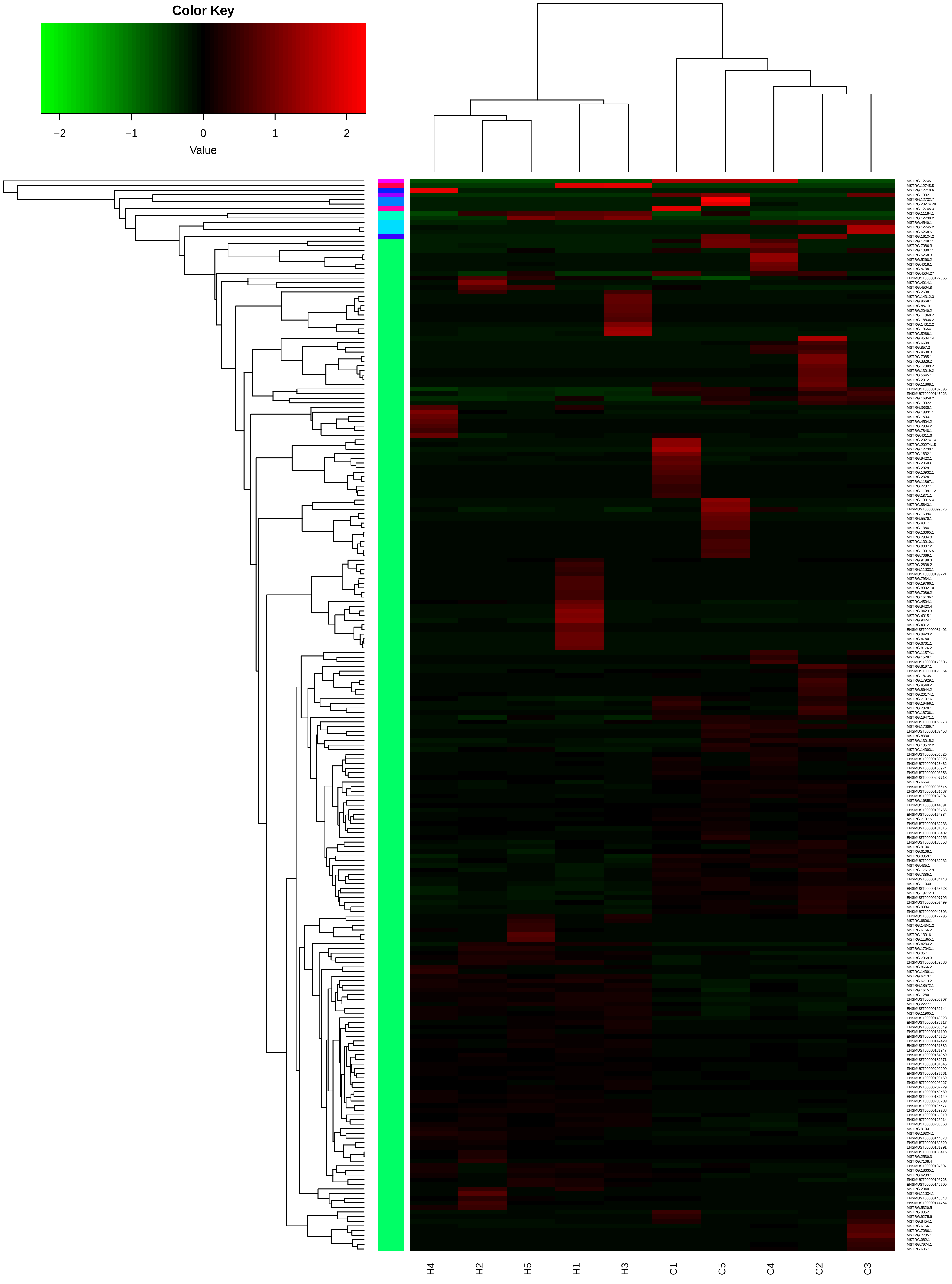

Heatmap of differentially expressed lncRNAs

Supplemental file 4. Heatmap of differentially expressed mRNAs and lncRNAs. H1-H5, high-fat diet-fed mice (n = 5); C1-C5, normal diet-fed mice (n = 5).
